# Growth Modulatory Role of Zinc in Prostate Cancer and Application to Cancer Therapeutics

**DOI:** 10.3390/ijms21082991

**Published:** 2020-04-23

**Authors:** Phuong Kim To, Manh Hung Do, Jin-Hyoung Cho, Chaeyong Jung

**Affiliations:** 1 Department of Anatomy, Chonnam National University Medical School, Gwangju 61469, Korea; tkphuong2609@gmail.com (P.K.T.); manhhung.cnsh@gmail.com (M.H.D.); 2 School of Medicine and Pharmacy, Tra Vinh University, Tra Vinh City 87000, Vietnam; 3 Department of Orthodontics, School of Dentistry, Chonnam National University, Gwangju 61186, Korea; jhcho@jnu.ac.kr

**Keywords:** zinc, prostate, prostate cancer, tumor growth, homeostasis

## Abstract

Zinc is a group IIB heavy metal. It is an important regulator of major cell signaling pathways in most mammalian cells, functions as an antioxidant and plays a role in maintaining genomic stability. Zinc deficiency leads to severe diseases in the brain, pancreas, liver, kidneys and reproductive organs. Zinc loss occurs during tumor development in a variety of cancers. The prostate normally contains abundant intracellular zinc and zinc loss is a hallmark of the development of prostate cancer development. The underlying mechanism of this loss is not clearly understood. The knowledge that excess zinc prevents the growth of prostate cancers suggests that zinc-mediated therapeutics could be an effective approach for cancer prevention and treatment, although challenges remain. This review summarizes the specific roles of zinc in several cancer types focusing on prostate cancer. The relationship between prostate cancer and the dysregulation of zinc homeostasis is examined in detail in an effort to understand the role of zinc in prostate cancer.

## 1. Introduction

Studies of zinc in plants have a long history and have been followed by animal and human studies [1]. Zinc is an essential cellular component that functions as an antioxidant and maintains genomic stability [2]. This biologically important metal ion is a constituent of more than 3000 proteins and is a cofactor for over 300 enzymes [3,4]. Zinc is required for normal growth. Its deficiency leads to abnormal growth that include improper brain development, prolonged wound healing and an impaired immune system [5,6]. Inadequate zinc uptake increases the risk of infections and is linked to aging-related symptoms, such as decreased immune competence, delayed wound healing and alteration of certain neurological and psychological functions [1].

Zinc is an essential mediator of cell proliferation and differentiation through the regulation of DNA synthesis and mitosis. Zinc also affects DNA repair pathways by regulating multiple intracellular signaling pathways and altering proteins involved in DNA maintenance. The effects of zinc on DNA polymerase were studied in both zinc-sufficient and zinc-deficient conditions over 40 years ago [7,8]. DNA polymerase activity was markedly lower in zinc insufficient rat embryos compared with that in rat embryos that developed in a zinc-sufficient condition. Another study demonstrated that DNA damage was caused by diminution of zinc in peripheral blood cells and that repletion of zinc protected from zinc-mediated DNA damage [9]. Low intracellular concentration of zinc has been associated with the increased expression of apyrimidinic endonuclease, which cleaves DNA at sites of damage [10]. Zinc in the form of ZnSO_4_ inhibits the repair of damaged DNA damage induced by hydrogen peroxide (H_2_O_2_) in K562 leukemia cells [11]. Withdrawal of zinc from PrEC prostate epithelial cells also stimulates breakage of single-strand DNA [12]. In addition, genes related to DNA damage response, including tumor protein p73 and MRE11, were downregulated in these cells, whereas the expression of p53 was increased. In murine fibroblasts, the addition of zinc can stimulate DNA synthesis and mitogenic signaling, whereas withdrawal of zinc reduces the secretion of growth hormone [13,14]. In Swiss 3T3 fibroblasts, ZnSO_4_ can reverse the inhibitory effect of diethylenetrinitrilopentaacetate (DTPA) on thymidine incorporation into DNA, suggesting that zinc stimulates cell growth by regulating cell cycle at the G1/S phase [15]. Intracellular zinc can block the G2/M transition in human bronchial epithelial cells by upregulating p53 and p21 activity [16]. These collective findings highlight the central role of zinc in the modulation of cell proliferation, mainly by affecting DNA synthesis. Therefore, zinc homeostasis plays a key role in the development of many diseases, in which the alteration of zinc is a common event.

## 2. Zinc Biology

**Figure 1:**
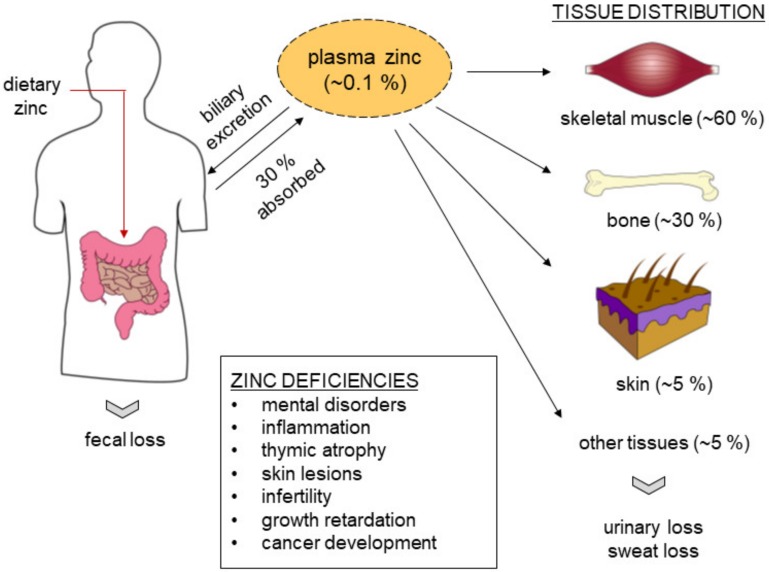
Zinc storage and distribution in the human body. The human body contains 2 to 3 g of zinc, which is absorbed by the duodenum and jejunum in the small intestine. Approximately 0.1% zinc is present in serum, 80% is loosely bound to albumin and approximately 20% is bound tightly to α2-macroglobulin. Approximately 60% of the zinc is stored in skeletal muscle, 30% in bone and approximately 5% in the skin and liver. The remaining zinc is distributed in other tissues, such as brain, kidneys, pancreas and heart. Zinc is excreted primarily through the gastrointestinal tract, with minor loss through urinary excretion.

**Figure 2:**
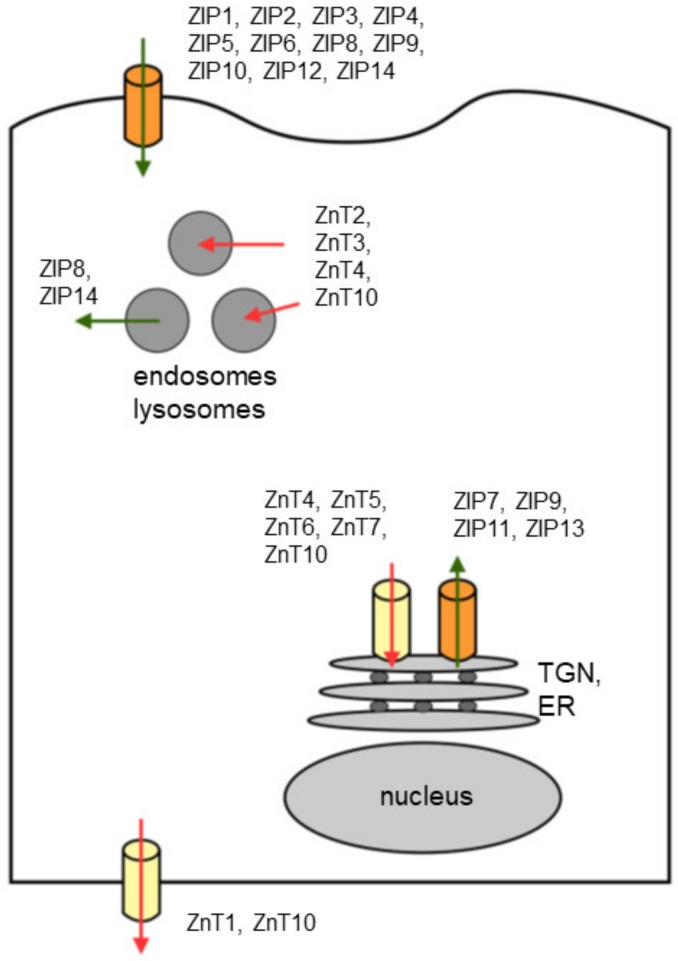
Zinc transporters and their subcellular localization. Subcellular localization of ZIP (green arrow) and ZnT (red arrow) is shown based on currently available information. The cytosolic zinc is mobilized into or out of different subcellular compartments, as indicated with arrows. Abbreviations are: TGN, trans-Golgi network; ER, endoplasmic reticulum.

The human body mass contains more than 2 g of zinc. Over 90% is distributed to most tissues, with only approximately 0.1% of this metal ion circulating in plasma [17,18]. Yet, this small amount of zinc plays an important role in maintaining homeostasis in the body. Zinc is stored in most organs and tissues with approximately 60% in skeletal muscle, 30% in bone and 5% in liver and skin and the remainder distributed in other tissues that include the brain, kidneys, pancreas and heart [19] (Figure 1). Excess zinc is primarily released through gastrointestinal secretion and endogenous excretion, with minor loss through urinary excretion. Although zinc is an essential trace element used by many enzymes and transcription factors, high concentrations are toxic to the cells. Cells adapt to overcome the toxicity by maintaining the balance of zinc uptake, intracellular storage and efflux [20]. In mammalian cells, intracellular zinc exists in two forms—a tightly or loosely bound form and an unbound form at very low concentrations as free Zn^2+^ ion [21]. Zinc binds avidly to metalloenzymes, metalloproteins and nucleoproteins and loosely to various proteins and amino acid ligands [22]. Many cells possess pico-molar levels of free zinc that is not bound to protein, which functions in cell control and cell-to-cell communication [23,24]. Therefore, strict regulation of the intracellular level of zinc is required for the maintenance of physiological conditions. A growing body of evidence suggests that both intracellular and compartmental zinc homeostasis is tightly controlled by the ZnT and ZIP families of transporter proteins. These crucial transporters are responsible for stabilizing intracellular zinc within cells [25]. The ZIP (SLC39A) family consists of 14 subtypes (ZIP1–14). They carry various metal ions including zinc into the cell cytoplasm from the lumen of organelles or across the membranes of cells (Table 1). The ZnT (SLC30A) family consists of 10 subtypes (ZnT1–10) that function in the efflux of cytoplasmic zinc into the lumen of organelles or into the intercellular space (Table 2). These transporters are either tissue specific or universally expressed in tissues depending on transporter subtype as summarized in Table 1 and Table 2 and Figure 2. Dysregulation or the malfunction of these transporters leads to various diseases and subsequent abnormal zinc-mediated metabolism is a common link in the development of most cancers.

## 3. Zinc and Zinc Transporters in Prostate Cancer

Zinc has long been known to be highly concentrated in prostate tissue—more than 10 times enriched compared to that in other soft tissues [92]. Analysis of frozen prostate tissues demonstrated lower levels of zinc in prostate carcinoma compared to those in normal prostate [93]. Zinc content of 1018, 1142 and 146 μg/g dry weight in normal prostate, benign prostatic hyperplasia (BPH) and prostate carcinoma tissue, respectively, was reported [94]. Zinc concentration in malignant prostate is approximately 10% to 25% lower than that of normal prostate [95]. Zinc contents in plasma are also significantly lower in prostate carcinoma (27%) and BPH (18%) compared to those in normal prostate samples [96]. A meta-analysis documented significantly lower levels of zinc in prostate cancers than in benign tumors and normal prostates [97]. Taken together, these findings indicate that the development of prostate malignancy is strongly associated with the reduction of intracellular zinc in malignant cells and the circulating level in plasma.

As the alteration of zinc homeostasis is controlled by cellular zinc transporters, many efforts have made to examine how intracellular zinc is regulated through zinc transporters [98,99,100]. Zinc trafficking requires specialized plasma membrane transporters. Their dysregulation causes abnormal growth of the prostate, including cancer. It is not well understood why prostate cancer cells depress cellular zinc levels. One proposed explanation is that the transformation of citrate producing normal cells to citrate-oxidizing malignant cells leads to the loss of the ability of the cells to accumulate zinc [95]. In addition, a genetic alteration in the expression of zinc transporters is associated with this metabolic transformation.

ZIP1, a member of the ZIP (SLC39) family, carries zinc or other metal ions from the extracellular space and/or intracellular organelles to the cytoplasm [2]. During prostatic cell transformation, the level of ZIP1 was remarkably decreased or absent compared to that in BHP or normal prostate [101]. Comparison of RWPE1 non-tumorigenic human prostate cells with its RWPE2 tumorigenic counterpart revealed the markedly lower zinc uptake in RWPE2 cells than in RWPE1 cells (33% decrease) and the downregulated expression of ZIP1 protein in RWPE2 cells [102]. Another comparison of the transgenic adenocarcinoma of the mouse prostate (TRAMP) model to wild type mice demonstrated the markedly lower zinc level and loss of ZIP1 expression in the prostate gland of TRAMP mice [103]. In LNCaP and PC3 prostate cancer cell lines, ZIP1 is the major zinc uptake transporter [104]. Forced expression of ZIP1 in these cells stimulated intracellular accumulation of zinc and inhibited cell proliferation due to increased apoptosis [26]. ZIP2 serves as another zinc uptake transporter across the plasma membrane and low levels of ZIP2 are restricted to a few tissues, including prostate and uterine glands [105]. Both ZIP2 and ZIP3 share a similar function with their ZIP1. Both were downregulated in malignant prostate cells and associated with the loss of zinc accumulation in tumor cells [106]. ZIP4 is also involved in zinc influx and is reduced in cancer tissues [107]. Despite the general reduction of ZIP proteins in prostate cancers, a correlation between the expression levels of most ZIP subspecies and cancer grade has not been established.

In contrast to the zinc influx protein family, zinc efflux transporters seem to be less involved in the transformation of the prostate gland and consequently have been less studied. In one study, ZnT1 expression was decreased or remained unchanged in prostate cancers than in BPH [108]. Decreased expression of ZnT4 was observed during the progression of prostate cancers, being under-expressed in both localized and metastatic prostate cancers compared to that in benign tissues [109]. ZnT4 expression was reportedly localized in intracellular vesicles and plasma membranes. At the RNA level, *ZnT1*, *ZnT9* and *ZnT10* were significantly upregulated in human prostate cancer tissues compared to those in adjacent normal tissues, implying that intracellular zinc is diminished through this upregulation of zinc output transporters [110]. *ZnT7* null-mutation in TRAMP mice was reported to accelerate the formation of prostate tumors compared to that in TRAMP mice retaining wild type *ZnT7* [111]. Expression of other zinc input transporters, including ZnT2, ZnT3, ZnT5, ZnT6 and ZnT8, has not been fully described and detailed studies are still ongoing. For now, there is not a clear understanding of zinc equilibrium.

Prostate specific antigen (PSA) is highly expressed in LNCaP cells. This can facilitate LNCaP cell invasion by degrading the extracellular matrix fibronectin and laminin glycoproteins [112]. Zinc strongly inhibited the enzymatic activity of PSA and suppressed the invasion of LNCaP cells, suggesting that zinc inhibits malignant prostate cancer cell invasion [113]. Physiological levels of zinc (0.25–0.5 µg/mL) inhibit nuclear factor-kappa B (NF-κB) activities by reducing RelA activity induced by tumor necrosis factor-alpha (TNF-α) and scaling down the expression of cellular inhibitors of apoptosis protein 2 (c-IAP2) in highly invasive androgen-independent DU145 and PC3 prostate cancer cell lines [114]. Furthermore, the zinc-reduced expression of vascular endothelial growth factor (VEGF), interleukin (IL)-6, IL-8 and matrix metalloproteinase-9 (MMP-9), which have been generally identified as pro-angiogenic and pro-metastatic molecules. Zinc can also diminish the expression of intercellular adhesion molecule-1 (ICAM1) to suppress tumor cell invasion and adhesion [115]. Homeobox B13 (HOXB13), a DNA-binding transcription factor, is overexpressed in castration-resistant prostate cancer and causes the zinc concentration to fall. This decrease subsequently stimulates cancer invasion and metastasis by promoting NF-κB signaling, through the reduction of NF-κB inhibitor (IκBα) [116]. HOXB13-mediated suppression of zinc is accomplished through the stimulation of the expression of the ZnT4 zinc efflux transporter but does not affect input transporters. These results indicate that the loss of intracellular zinc could enhance HOXB13 expression in prostate cancer, leading to the stimulation of the NF-κB signaling pathway to promote prostate cancer metastasis. Zinc also affects the activity of urokinase-type plasminogen activator (uPA) and aminopeptidase N (AP-N) to suppress the invasion and metastasis of PC-3 prostate cancer cells [117]. The collective findings strongly indicate that excess quantities of zinc negatively regulate prostate cancer cell growth, invasion and metastasis.

## 4. Zinc and Zinc Transporters in Other Cancers

Whereas serum zinc levels are low during breast cancer development [118,119], biopsies from breast cancer patients have revealed significantly higher zinc levels compared with those in normal breast tissues [120,121,122]. Correspondingly, the expression of zinc transporters, including ZIP6, ZIP7 and ZIP10, were positively correlated with the risk of breast cancer [123]. The involvement of ZIP6 in longer relapse free survival and prolonged survival of breast cancer patients with ductal carcinoma invasion has been documented [124]. Knockdown of *ZIP6* in MCF-7 breast cancer cells can increase cell survival in hypoxic environments [125,126]. ZIP6 also reportedly promotes breast cancer cell invasion and metastasis, together with the high expression of E-cadherin [127,128]. Upregulation of ZIP7 was reported in high risk breast cancer and was linked to a poor prognosis [129]. ZIP6 expression was positively correlated with estrogen receptor (ER) and correlated with aggressive breast cancer with promoted metastasis [130,131]. More than 70% of breast cancer cells are characterized as ER positive (ER+) and anti-estrogen compounds are among the main therapeutic drugs for ER+ breast cancer cells. Unfortunately, the efficacy of the anti-estrogen drug tamoxifen for malignant breast cancer is limited due to the emergence of estrogen-independent breast cancers [132,133]. ZIP6 has been associated with higher zinc levels in breast tumor cells compared with those in normal breast cells and anti-estrogen compounds can reduce cellular zinc pools [134]. Zinc and ZIP7 was increased in tamoxifen resistance MCF-7 cells, which enhanced growth factor activity and induced cancer cell growth and invasion [135]. Suppression of ZIP7 can repress epidermal growth factor receptor signaling, which subsequently reduces tumor cell growth and prevents the acquisition of breast cancer resistance to tamoxifen. These results suggest that abnormal regulation of ZIP6 and ZIP7 and intracellular zinc contents are strongly involved in breast cancer cell proliferation and migration. ZIP10 expression was reportedly significantly higher in highly invasive and metastatic breast cancer cells (MDA-MB-231 and MDA-MB-435S) than in less metastatic breast cancer cells (MCF7, T47D, ZR75-1 and ZR75-30). Accordingly, ZIP10 was associated with lymph node metastasis of breast cancer; the suppression of ZIP10 can inhibit the migration of breast cancer cells [49].

Dysregulation of zinc and zinc transporters have also been considered as the major factors for progression of pancreatic cancer. ZIP3 and ZIP4 are two well-studied transporters that display altered expression in pancreatic tumor tissues. One study described the loss of zinc in ductal and acinar epithelium of pancreatic cancers in which ZIP3 expression was downregulated compared with that in normal pancreatic epithelium [136]. ZIP4 is reportedly overexpressed in 94% of pancreatic adenocarcinomas compared with that in surrounding normal tissues [137]. The forced expression of ZIP4 increased intracellular zinc levels, increased cell proliferation and dramatically increased tumor volume in nude mice, suggesting that zinc availability and aberrant ZIP4 expression might be essential for pancreatic tumor growth. In esophageal cancers, markedly lower plasma zinc levels as compared to the levels in esophagitis and normal groups were described [138]. Another study described the overexpression of ZIP5 in esophageal squamous cell carcinoma compared to that in normal tissue and that knockdown of ZIP5 reduced cell proliferation, migration and invasion due to the suppression of COX2 and cyclin D1 [139]. In NCI-H358 lung cancer cells, ZIP1, ZIP4, ZIP7 and ZIP10 were all elevated, with ZIP4 expression being highest. Although the expression of ZnTs was generally low, ZnT7 and ZnT9 were significantly overexpressed in lung tumor tissues [140]. In bladder cancers, ZnT1 was overexpressed and suppression of ZnT1 led to the inhibition of the proliferation, migration and invasion in BIU87 bladder cancer cells [141]. In hepatocellular cancer, zinc was lost in 55% of hepatocellular cancers [142]. Increasing numbers of reports suggest that the abnormal regulation of zinc is involved in many cancers, including prostate, breast and pancreatic cancers. The pattern of zinc alteration is somewhat tissue specific and zinc generally induces inhibition of cancer cell growth by targeting the intrinsic apoptotic pathway. Although the mechanisms of how zinc dysregulation drive cancer development are not very well established, the expression of zinc transporters are commonly altered in multiple cancers and these transports have been implicated in this process.

## 5. Zinc as an Agent for Treatment of Prostate Cancer

The human body contains over 2 g of zinc with the highest content present in the prostate [143]. The total cellular zinc concentration for most mammalian cells typically ranges from 100 to 500 μM [144,145]. However, zinc is concentrated in epithelial cells in the peripheral zone of the prostate in the range of 800 to 1500 μM [146]. Only limited bioavailable free zinc is available [2,93,95,101,147,148]. The distribution of zinc in the cells is approximately 30% to 40% in the nucleus, 50% in the cytoplasm, with the remainder in the cell membrane [149,150]. At the same time, the total intracellular zinc (0.2–1 mM) is divided into three pools, including tightly bound zinc as an immobile and unreactive pool, loosely bound zinc and a reactive pool of free zinc ion. Approximately 90% of cytoplasmic zinc is bound to immobile macromolecules, mostly proteins, with 10% bound to mobile low molecular weight ligands [151]. As previously mentioned, the content of zinc in prostate carcinoma is much lower than that in normal prostatic epithelial cells [93]. Zinc deficiency in prostate cancer cells has led to the central dogma that the supplementation of zinc may contribute to the prevention of prostate cancer as well as halting cancer malignancy. The re-introduction of physiological levels of zinc into cancer cells has yielded diverse results that have challenged the interpretation of the biologic functions of zinc. Low doses of zinc may not reach the biological threshold, while at higher doses zinc may become ineffective due to its toxicity [152]. Therefore, most therapeutic studies have been done using excessive amounts of zinc due to the aforementioned cellular distribution of zinc. Table 3 summarizes several prostate cancer therapeutic studies previously performed using various doses of zinc in vitro and in vivo. Effective growth inhibition for LNCaP cells was accomplished at 100 ng/mL zinc, whereas a higher concentration (700 ng/mL) was required to show similar growth inhibition in PC-3 cells [153]. Zinc-mediated growth inhibition was accomplished through the induction of apoptosis, arrest of cells in the G2/M phase of the cell cycle and zinc-mediated increased expression of p21^Waf1/Cip1/Sdi1^. Zinc treatment also released cytochrome c from mitochondria to cytosol, activated caspase 3 and 9 and cleaved nuclear poly (ADP)-ribose polymerase (PARP), which activated apoptosis in malignant prostate cancer cells [154,155]. The growth of most prostate cancer cells, including LNCaP, DU145 and PC-3, can be inhibited by the addition of ZnSO_4_ in a range from 200 to 600 μM [156,157]. Zinc also inhibits hypoxia inducible factor-1 alpha (HIF1α) expression and its activity to repress cancer stimulating pathways, such as VEGF and Bcl2 [158]. In addition, zinc contributes to the truncation of the Krebs cycle and inhibition of citrate oxidation, which further prevents cancer cell growth and proliferation, as well as inhibiting the invasion and migration of cancer cells [95]. 

Many in vivo studies have verified that zinc efficiently suppresses prostate cancer tumors. When zinc was administered to PC-3 cell-bearing nude mice by osmotic pumps for 4 weeks, tumor growth was markedly reduced with the intracellular accumulation of zinc, followed by the elevated expression of the apoptosis-induced protein Bax/Bcl-2 [162]. Direct injection of 200 to 600 μM zinc into mice-bearing PC3 tumors halted growth of the tumors and subsequently extended the survival of the animals, with no detectable cytotoxicity to other tissues [157]. Furthermore, the intraperitoneal injection of TRAMP-C2 bearing mice with 10 mg/kg body weight zinc led to the remarkable decrease of tumor volume with the reduced expression of androgen receptor [164]. A study using various doses of zinc in TRAMP mice as an attempt to investigate the chemopreventative potential of zinc showed that a zinc-sufficient diet protected tumor development in the mice [163]. Administration of 100 ppm (or 0.01%) of zinc in drinking water for 20 weeks reversed the various effects induced by carcinogenic N-methyl-N-nitrosourea combined with testosterone [165]. These effects included tumor formation, phosphatase activity and expression of p53, Bcl-2 and caspase-3 on the dorsolateral prostate of rats, implicating zinc in protecting from carcinogen-induced tumor progression. In a clinical study involving nearly 700 patients with prostate cancer, adequate uptake of zinc was associated with a reduced risk of prostate cancer [166]. A study involving 525 men with prostate cancer in Sweden also showed that a high zinc diet reduced the risk of prostate cancers [167]. Although the majority of studies supported the hypothesis that zinc intake by cancer cells can prevent growth of the cells [146,153,168], other studies reported that zinc supplementation was neutral or detrimental to prostate cancer progression [169,170]. For example, evaluation of the influence of zinc treatment on cancer risk in the VITamins And Lifestyle (VITAL) cohort revealed that 10 years consumption of a zinc diet did not reduce prostate cancer risk, while the intake of an average intake of >15 mg/day of zinc decreased risk of advanced prostate cancer [171]. A large epidemiological study performed by the United States National Cancer Institute suggested that supplemental zinc intake at doses of 100 mg/day for 14 years was not associated with prostate cancer risk, although a higher risk of advanced prostate cancer was evident in a small group of individuals [172].

## 6. Perspective

For many years, extensive investigations to decipher the precise role(s) of zinc ion have been conducted in both normal and cancer cells. Zinc is an essential component for all forms of life and is a crucial trace element required for the activity of more than 300 enzymes. Over 2000 zinc-finger transcription factors are deeply involved in growth-modulating cell signaling pathways. Consequently, zinc deficiency is responsible for the development of various diseases, such as abnormal body growth, immune dysfunction, diabetes and cancers. Loss of zinc has been documented in patients diagnosed with a variety of cancer types, including prostate cancer, hepatocellular cancer, pancreatic cancer, lung cancer, ovarian cancer, esophageal squamous cell carcinoma and breast cancer. Among all soft tissues, the prostate is most enriched in zinc. Decrease in intracellular zinc is a feature of prostate cancer development and even progression to malignancy. The diverse functions of zinc in prostate cancer include inhibition of cell proliferation by induction of the cell cycle and the inhibition of cell migration and invasion. Zinc and zinc derivatives have been extensively studied to test the hypothesis that therapies that lead to the accumulation of zinc in cancer cells effectively inhibit the proliferation of these cells. A great deal of experimental evidence supports the idea that zinc derivatives and zinc supplements are able to suppress the proliferation, migration and invasion of prostate cancer cells. Moreover, the appropriate intake of zinc into cancer cells can reduce the risk of prostate cancer. However, the efficacy of zinc provided in any form seems to be limited mainly due to the inability of cancer cells to import excessive zinc from the extracellular milieu. Hence, many ongoing studies have explored the relationship between zinc and functional zinc transporters, such as ZIP1, which is lost or decreased in many prostate cancers. There are also many conflicting results concerning the curative and preventative roles of zinc in prostate cancers. Several epidemiologic studies have suggested that zinc supplementation may increase the risk of advanced prostate cancer. The inconsistency in data concerning dietary zinc supplementation and the zinc-related impact on prostate cancer prevention and treatment has cast suspicion on zinc-mediated therapies. This issue needs to be extensively investigated. Understanding the mechanism by which zinc is lost during prostate malignancy and detailed information underlying the protective role of zinc in prostate cancer will help to address its importance in the malignancy and progression of prostate cancer and thus the value of zinc in prostate cancer prevention and therapy.

## Figures and Tables

**Table 1:** Human ZIP proteins.

Protein	Gene Locus	Tissue/Cell Distribution	Subcellular Localization	References
ZIP1/ZIRTL	1q21	wide spread	plasma membrane,	[26,27,28]
ZIP2/Eti-1/6A1	14q11.1	wide spread	plasma membrane	[29,30,31]
ZIP3	19p13.3	wide spread, predominant in testis	plasma membrane	[32,33]
ZIP4	8q24.3	gastrointestinal tract, kidney, hippocampal neurons	plasma membrane	[34,35,36]
ZIP5/LZT-Hs7	12q13.13	pancreas, kidney, liver, stomach, intestine	plasma membrane	[37,38,39]
ZIP6/LIV1	18q12.1	widespread	plasma membrane	[40,41]
ZIP7/HKE4	6p21.3	widespread	ER, Golgi, intracellular vesicles	[42,43,44]
ZIP8/BIGM103/LZT-Hs6	4q22-q24	widespread, predominant in pancreas	plasma membrane, lysosomes, endosomes, mitochondria	[45,46]
ZIP9	14q24.1	widespread	plasma membrane, trans-Golgi (TGN)	[47,48]
ZIP10/LZT-Hs2	2q33.1	brain, liver, erythroid, kidney	plasma membrane	[40,49,50,51]
ZIP11	17q25.1	testis, digestive system	TGN, cytoplasm and nuclei	[52,53]
ZIP12	10p12.33	brain, lung, testis, retina	plasma membrane,	[54,55]
ZIP13	11p11.12	widespread	intracellular vesicles, Golgi	[56,57]
ZIP14	8p21.2	widespread	plasma membrane, endosomes	[58,59,60,61,62]

**Table 2:** Human ZnT proteins.

Protein	Gene Locus	Tissue/Cell Distribution	Subcellular Localization	References
ZnT1	1q32.3	widespread	plasma membrane	[25,63,64]
ZnT2	1p35.3	mammary gland, prostate, retina, pancreas, small intestine, kidney	plasma membrane, endosomes, lysosomes, secretory vesicles and mitochondria	[65,66,67,68]
ZnT3	2p23.3	brain, testes, pancreas	synaptic vesicles	[69,70,71,72]
ZnT4/ Dri27	15q21.1	widespread, predominant in mammary gland, placenta, prostate, brain and kidney	plasma membrane, endosomes, secretory vesicles	[73,74,75]
ZnT5/ ZTL1	5q13.1	widespread, predominant in pancreas, liver. kidney	TGN, plasma membrane	[76,77,78]
ZnT6	2p22.3	widespread	TGN, unknown vesicles	[79,80]
ZnT7	1p21.2	widespread, enriched in stomach, prostate, retina, pancreas, testis and muscle	Golgi, unknown vesicles	[81,82,83]
ZnT8	1q41	pancreas, thyroid, adrenal gland, testis	secretory vesicles	[84,85,86]
ZnT9/ C4orf1	4p13	widespread	cytoplasm, nucleus	[87,88]
ZnT10	1q41	brain, retina, liver	endosomes, endosomes, plasma membrane	[89,90,91]

**Table 3:** Various strategies for prostate cancer therapeutics with zinc.

Cells	Animals	Zinc Dosages	Delivery	Effects	References
PC3, LNCaP	in vitro	up to 1 µg/mL ZnSO_4_	culture media	Inhibition of cell growth: induction of apoptosis by G2/M arrest and increase of p21^Waf/Cip1/Sdi1^ expression	[153,154,155]
PC3, LNCaP	in vitro	50–150 μM zinc acetate	matrigel	Inhibition of cell invasion: Suppression of PSA and uPA activities	[113,159]
PC-3, DU145	in vitro	0.06–0.55 μg/mL ZnSO_4_	culture media	Inhibition of cell metastasis by regulation NF-κB and c-IAP2 activities; stimulation of AP-1; suppressed expression of VEGF, IL-6, IL-8 and MMP-9	[114,115]
PC3^ZIP1^	in vitro; C.B.17 SCID mice	1.5 μg/mL ZnSO_4_; 2000 ppm ZnSO_4_	culture media; drinking water	Overexpression of ZIP1 reduced cell growth and invasion by Inhibition of NF-κB activity	[114,115,160]
PC3	NOD/SCID mice	200 µL of 3 mM zinc acetate	intratumoral injection	Inhibition of tumor growth enhancement of animal survival	[157]
PC3	NOD/SCID mice	3–20 mg/kg ZnCl_2_	intraperitoneal injection	No effects on xenograft tumor cell growth	[161]
PC3	nude mice	ZnSO_4_ (30–45 μg/day) for 28 days	osmotic pumps	Inhibition of tumor growth by increased Bax/Bcl-2 protein expression	[162]
Transgenic prostate cancer	TRAMP mice	0.85, 30, or 150 ppm zinc carbonate (52.1% Zn) for 22 weeks	pellet	Increased tumor weights upon deficient or high zinc uptake	[163]
TRAMP-C2	C57BL/6 mice	10 mg/kg ZnCl_2_ for 2 weeks	intraperitoneal injection	Repressed tumor growth and androgen receptor expression	[164]
MNU and testosterone-induced PIN	Sprague Dawley rat	100 ppm ZnCl_2_ for 20 weeks	drinking water	Reverse effects on MNU and testosterone-mediated PIN	[165]

PSA, prostate specific antigen; uPA, urokinase-type plasminogen activator; c-IAP2, cellular inhibitors of apoptosis protein 2; TRAMP, transgenic adenocarcinoma mouse prostate, MNU, N-methyl-N-nitrosourea; PIN, prostatic intraepithelial neoplasia.
